# Detection of Stress Levels from Biosignals Measured in Virtual Reality Environments Using a Kernel-Based Extreme Learning Machine

**DOI:** 10.3390/s17102435

**Published:** 2017-10-24

**Authors:** Dongrae Cho, Jinsil Ham, Jooyoung Oh, Jeanho Park, Sayup Kim, Nak-Kyu Lee, Boreom Lee

**Affiliations:** 1 Department of Biomedical Science and Engineering (BMSE), Institute of Integrated Technology (IIT), Gwangju Institute of Science and Technology, Gwangju 61005, Korea; dongrae16@gist.ac.kr (D.C.); trueham@gist.ac.kr (J.H.); jooyoungoh@gist.ac.kr (J.O.); 2 Research Institute of Industrial Technology Convergence, Korea Institute of Industrial Technology, Ansan 15588, Korea; jeanho@kitech.re.kr (J.P.); sayub@kitech.re.kr (S.K.); nklee@kitech.re.kr (N.-K.L.)

**Keywords:** virtual reality (VR), kernel-based extreme learning machine (K-ELM), heart rate variability (HRV), autonomic nervous system (ANS)

## Abstract

Virtual reality (VR) is a computer technique that creates an artificial environment composed of realistic images, sounds, and other sensations. Many researchers have used VR devices to generate various stimuli, and have utilized them to perform experiments or to provide treatment. In this study, the participants performed mental tasks using a VR device while physiological signals were measured: a photoplethysmogram (PPG), electrodermal activity (EDA), and skin temperature (SKT). In general, stress is an important factor that can influence the autonomic nervous system (ANS). Heart-rate variability (HRV) is known to be related to ANS activity, so we used an HRV derived from the PPG peak interval. In addition, the peak characteristics of the skin conductance (SC) from EDA and SKT variation can also reflect ANS activity; we utilized them as well. Then, we applied a kernel-based extreme-learning machine (K-ELM) to correctly classify the stress levels induced by the VR task to reflect five different levels of stress situations: baseline, mild stress, moderate stress, severe stress, and recovery. Twelve healthy subjects voluntarily participated in the study. Three physiological signals were measured in stress environment generated by VR device. As a result, the average classification accuracy was over 95% using K-ELM and the integrated feature (IT = HRV + SC + SKT). In addition, the proposed algorithm can embed a microcontroller chip since K-ELM algorithm have very short computation time. Therefore, a compact wearable device classifying stress levels using physiological signals can be developed.

## 1. Introduction

Virtual reality (VR) involves creating and implementing a simulated, realistic, three-dimensional environment [1]. In other words, diverse virtual environments can be constructed in limited spaces by generating realistic images, sounds, and other sensations. Since environments generated by VR devices are similar to the real world, they have been used in various fields, especially as treatment options in hospitals. For example, VR devices have been used for social-adaptation training for social phobias, as well as for treating post-traumatic stress disorder (PTSD) [2]. In addition, many researchers have utilized VR devices during their experiments to create environments and observe the corresponding responses [3,4,5]. For instance, electroencephalogram (EEG) signals were measured in a VR environment, which are composed of three different traffic light situations (red, green, and yellow), and EEG signals were well discriminated according to the traffic light color [6]. In another study, the anxiety was triggered by VR environment using stressful job interview situation and analyzed the changes in the cardiovascular activity [7]. As a result, the cardiovascular change of people who had been trained in VR environment was less than that of untrained people. Taken together, the physiological signals seem to be well changed and modified with artificial VR environment.

Generally, stress is induced by physical, mental, or emotional tensions and causes changes in the body’s response. In addition, since VR environment can trigger unfamiliar stimuli for subjects, several phenomena such as the anxiety, mental concentration and nausea are regarded as stress in a broad sense. Especially, physiological changes can be caused by unfamiliar external environments and psychological changes [8]. Among the factors that can induce physiological changes, in particular, the autonomic nervous system (ANS) is important in regulating the functions of the internal organs and maintaining homeostasis, as well as human physiological activities [9]. Also, external factors including stress can affect ANS function, so that physiological phenomena and changes could diversely appear according to the levels of perceived stresses [10].

The ANS, when affected by stress stimuli, secretes stress hormones such as cortisol and adrenaline within the blood vessels. This causes the activation of sympathetic nerves and the inactivation of parasympathetic nerves [11]. As the result, physiological responses can appear in the body [12]. For example, there is an evidence that cognitive loads can affect the cardiac function [13]. Given that heartbeats depend on ANS activity, cardiac activity after tasks with cognitive loads can also be related to ANS changes.

Heart activity, skin sweating and skin temperature are known to be regulated by the ANS, so biosignals related to such activities (photoplethysmograms (PPG), electrodermal activity (EDA) and skin temperature (SKT)) could provide insights on ANS activity.

The PPG signal is measured from the finger, and reflects the change in blood volume in the peripheral blood vessels [14]. Since the changes in blood volume are associated with cardiac activity, the peak positions of the PPG signal are similar to the R-peak positions of QRS complex in an electrocardiogram (ECG) [15].

The heart rate variability (HRV) can be calculated from the intervals between the peaks of the PPG signal [16]. The HRV has been used to investigate changes in the ANS, as well as diagnosing heart disease. In particular, it is noteworthy that several HRV parameters (e.g., the ratio of high-frequency and low-frequency (HF/LF) powers) are associated with the activities of sympathetic/parasympathetic nerves [17].

The EDA is an electrical signal measuring continuous skin-conductance (SC) changes [18]. In general, changes in SC are associated with sweat gland activity. Whenever sweat glands secrete sweat through the pores, SC peaks are generated. In particular, the amplitudes and frequency of SC peaks are related to the activation of the sudomotor nerve, which is part of the ANS [19].

Lastly, changes in SKT are also associated with changes in the ANS. For instance, it is known that the combined inhibition of dopamine (DA) and norepinephrine (NE) reuptake by the activity of sympathetic nerves improves exercise performance and increases body temperature [20]. Taken together, the three physiological signals (PPG, EDA, and SKT) are associated with the activities of the ANS. Considering that ANS changes can be induced by stressful tasks with cognitive loads, we can postulate that the features obtained from measured signals can reflect not only changes in the ANS, but also the stress levels [17,18].

Recently, mobile healthcare system has been studied for detecting stress levels using various wearable sensors measuring the physiological signals [21,22]. The newest developed wearable sensors can continuously measure the physiological signals including PPG, EDA, and SKT, so that the users can check their health state without any inconvenience [23]. Mobile devices such as Galaxy gear and Apple iWatch are appropriate to be utilized in this field. Nowadays, most mobile device has high-performance microprocessor. With advances in engineering technology, the performance of microprocessors embedded in mobile devices has been improved and complex mathematical problem can be calculated as well. Eventually, classification methods that require the complex computation can work with the microprocessor embedded in mobile device.

Automatic classification methods using features extracted from biosignals have been developed [24,25,26]. In general, conventional neural network algorithms are used to estimate optimal boundaries for separating distinct classes and also to perform iterative processes to obtain the optimized weights and biases of each layer. However, a long and complex iterative process is required to utilize large datasets, which include extensive biological information [27]. Moreover, it is difficult to be operated on microprocessor having limited resources. To solve this problem, the extreme learning machine (ELM) was developed.

Since the ELM is based on single-hidden–layer feedforward neural networks (SLFNs), and randomly selects the input weights and biases for the hidden layer, it has low computational complexity as well as high classification accuracy [28]. In addition, the kernel-based extreme learning machine (K-ELM), an extended version of the ELM, is more robust than the ELM, with smaller computation time [27,29]. In other words, the K-ELM uses fewer resources while showing high performance compared to other neural networks. Therefore, K-ELM algorithm can work in a microcontroller chip with a small memory.

In this study, a stressful task with cognitive loads was designed to measure stress-related biosignals in a VR environment. The task was composed of five sequential sessions with varying stress levels: baseline, mild stress, moderate stress, severe stress, and recovery. During this task, physiological signals (PPG, EDA, and SKT) were measured simultaneously. Then, we classified these five different states by combining K-ELM and the features obtained from the three physiological signals. Additionally, we evaluated whether the calculated features reflect intended stress levels, and compared the performances of the conventional machine learning algorithms with those of the proposed algorithm.

## 2. Materials and Methods

### 2.1. Data Acquisition

#### 2.1.1. Participants

Twelve healthy subjects (six females, six males) voluntarily participated in the study. The average age of the subjects was 27.5 years (standard deviation (SD): ±3.18). None of the subjects had any experience with the VR equipment. In addition, they had not taken any medicine, etc. that could affect the result of this study. Before participating in the experiment, the subjects were fully aware of the purpose and procedure of our research. This study was approved by the Institutional Review Board of the Gwangju Institute of Science and Technology (GIST).

#### 2.1.2. Stress-Inducing Task in VR Environments

The participants performed a stress-inducing task with a cognitive load: arithmetic subtraction. The task proceeded in configurable VR environments using the commercial Gear VR device (Samsung Electronics, Inc., Suwon, Korea). Our task was composed of five sessions, including three with different levels of stress: mild stress (MIS-S), moderate stress (MOS-S), and severe stress (SES-S). The other two sessions were the baseline session (BA-S) and the recovery session (RE-S).

To acquire suitable VR videos, we investigated the YouTube website (https//www.youtube.com) and initially selected nine VR videos. Then, 22 people (who did not participate in the main experiment) scored the nine VR videos to assess how much stress they triggered; finally, three VR videos were selected. According to the scores, we assigned the three videos to the mild, moderate, and severe stress-inducing sessions.

During the MIS-S, we provided a relatively static VR environment, consisting of a monotonous landscape with the sound of waves on the beach. At the same time, the subject subtracted double-digit numbers from four-digit numbers (e.g., 1000 − 17). In the MOS-S, we provided a relatively dynamic VR environment consisting of a car-racing situation on a rainy day, while similar arithmetic tasks were performed at the same time. Lastly, the SES-S was composed of VR stimuli that could induce a sensation of fear: a dark, dingy room where a guard on patrol appears while a frightening background sound is heard. Again, similar arithmetic tasks were conducted simultaneously. To avoid the familiarity of arithmetic calculation in each stress-inducing session, we randomly changed the numbers in each session [3].

After the experiment, we conducted two questionnaire surveys to evaluate how much stress was induced: the state-trait anxiety inventory (STAI) Y-1 and the visual analogue scale (VAS). The STAI Y-1 questionnaire consists of 20 questions and is often used to assess anxiety states [29]. The VAS is a subjective assessment of a stress-inducing task and is scored from 0 (lowest stress) to 10 (highest stress). Each subject filled out a questionnaire for each stress-inducing session [30].

#### 2.1.3. Experimental Procedure

**Figure 1:**
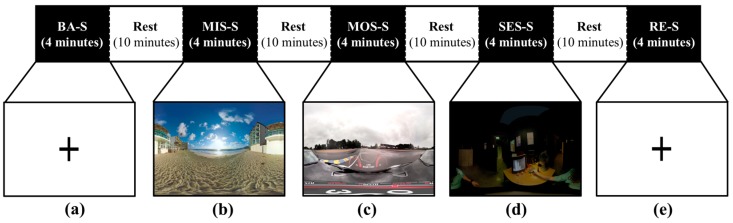
Total experiment flow: (**a**) Cross mark in the baseline session; (**b**) mild-stress session with beach scenery; (**c**) moderate-stress session with a car-racing situation; (**d**) severe-stress session with a dark, dingy room; (**e**) cross mark in the recovery session.

We performed a stress-inducing experiment with five sessions: BA-S, MIS-S, MOS-S, SES-S, and RE-S. In BA-S and RE-S, a black cross mark was displayed on a white background on the monitor. The difference between BA-S and RE-S is their order during the experiment. The flow of our experiment is shown in Figure 1. The length of each session is four minutes. Whenever each session finished, the subject rested for 10 min to reduce the effect of the previous session. All experiments were performed while the subjects were seated in a comfortable chair.

Three physiological signals were measured from each subject during the experiment: a photoplethysmogram (PPG), electrodermal activity (EDA), and skin temperature (SKT). The physiological signals were obtained using the Biopac PPG100C, Biopac EDA100C, and UIM100C systems (Biopac System, Inc., Goleta, CA, USA). The PPG sensor was attached to the index finger, EDA sensors were attached to the middle and ring fingers, and the SKT sensor was attached to the thumb of the non-dominant hand. Each physiological signal was sampled at 400 Hz according to the manufacturer’s instructions, using the Biopac Acknowledge 4.2.0 software (Biopac System, Inc.). Figure 1 shows the overall experimental procedures.

### 2.2. Feature Preparation

#### 2.2.1. Heart-Rate Variability (HRV)

An HRV analysis was performed using the PPG signal of each subject. We assumed that the frequency range of the motion artifact was less than 0.1 Hz, the frequency range of the respiration was 0.15 Hz to 0.4 Hz, and the frequency range of the heartbeat was around 1 Hz [16]. Therefore, we applied a band-pass filter (0.5 Hz to 4 Hz) to remove unnecessary signals and enhance the heartbeat. After that, a peak-detection algorithm [31] was used to calculate the intervals between the PPG peaks. The difference in the detected peaks is referred to as the normal-to-normal (NN) interval and indicates heartbeat fluctuation [16,32,33]. We divided the HRV features into three parts: time-domain, frequency-domain, and nonlinear measures. The time-domain measures quantify changes in the NN interval through statistical methods and can indicate the overall change of the heartbeat in the time domain. Time-domain features were composed of ${\mathrm{HR}}_{\mathrm{avg}}$, ${\mathrm{NN}}_{\mathrm{avg}}$, SDNN, SDSD, RMSSD, pNN20, and pNN50, defined in Table 1.

Frequency-domain measures can be obtained from the power spectrum calculated from the NN tachogram. The NN tachogram can be obtained by interpolating the unevenly sampled NN intervals [17]. The power-spectrum density (PSD) of the NN tachogram was made using autoregressive (AR) spectrum modeling in this study [34]. Eventually, the low-frequency (LF) and high-frequency (HF) powers can be obtained by calculating the area under the curve from 0.04 to 0.15 Hz and from 0.15 to 0.4 Hz, respectively. Frequency-domain features consisted of ${\mathrm{LF}}_{\mathrm{normal}}$, ${\mathrm{HF}}_{\mathrm{normal}}$, and LF/HF, as exhibited in Table 1.

Lastly, nonlinear measures are as follows: approximate entropy (ApEn) [35], sample entropy (SampEn) [36], SD1, SD2, and SD1/SD2 [37]. ApEn can indicate the irregularity or complexity of the NN interval, and depends on the embedded dimension of vector m and threshold r. SampEn is an algorithm compensating for the disadvantages of ApEn. Because ApEn inherently contains a bias with regard to regularity, the regularity will be proportionated to the number of self-matches.

**Figure 2:**
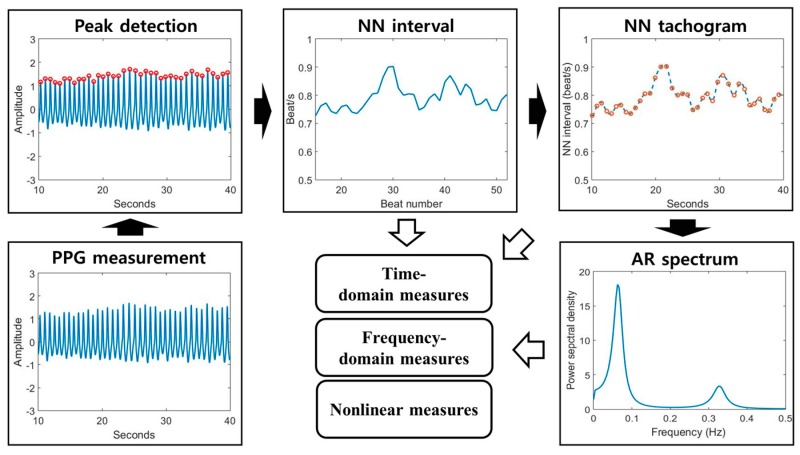
Block diagram of HRV analysis. The HRV analysis procedure includes the following steps: PPG measurement, peak detection, NN interval calculation, NN tachogram estimation, and autoregressive (AR) spectrum analysis. After these steps, we finally obtain the time, frequency, and non-linear features.

However, since SampEn does not include a self-match process, it is relatively trouble-free and stable. Therefore, the result of SampEn analysis is much more robust than the result of ApEn analysis [35]. Poincaré analysis is used to quantify self-similarity and evaluate the dynamics of a system—a Poincaré plot is a graph in which each NN interval is plotted against the next NN interval; a detailed calculation process was indicated in [37]. SD1 is the standard deviation of the data perpendicular to the axis of line-of-identity. SD2 is the standard deviation of the data along the axis of line-of-identity. Figure 2 shows the overall HRV analysis methods. All indicators of HRV was calculated by our own analysis system written in MATLAB.

#### 2.2.2. Skin Conductance (SC)

The SC signal indicates the electrodermal activity (EDA) measured by a non-invasive electrode on the skin. The SC signal consists of two types of signals: slow-varying tonic activity and fast-varying phasic activity. 

**Figure 3:**
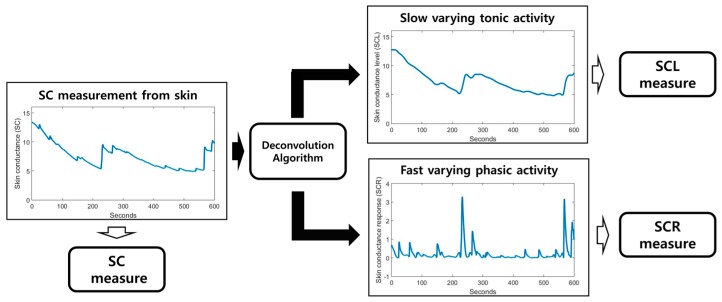
Block diagram for SC analysis. The SCL and SCR can be obtained by deconvoluting the SC signal.

They are called skin-conductance level (SCL) and skin-conductance response (SCR), respectively. SCL is a baseline level; it changes in the absence of any environmental events. On the other hand, SCR changes when environmental events occur. Furthermore, it is also known that changes in SCR are associated with activity of the sudomotor nerves related to the sweat glands [18]. In this context, we applied the decomposition algorithm, which is a deconvolution algorithm [38], to divide the SC signal into SCL and SCR. Several features are related to the SC, and we utilized the following features in our analysis: ${\mathrm{SC}}_{\mathrm{avg}}$, ${\mathrm{SCL}}_{\mathrm{avg}}$, ${\mathrm{SCL}}_{\mathrm{slope}}$, ${\mathrm{SCR}}_{\max}$, and ${\;\mathrm{SCR}}_{\mathrm{peak}}$ (Table 1). The decomposition procedure was performed using the Ledalab 3.4.9 toolbox written in MATLAB [39]. Figure 3 shows a simple flowchart of SC decomposition.

#### 2.2.3. Skin Temperature (SKT)

It is known that continuous stress can trigger an increase in body temperature by affecting the ANS [18]. Furthermore, the activity of sympathetic nerves improves exercise performance and increases the body temperature [40]. We selected SKT features, e.g., ${\mathrm{SKT}}_{\mathrm{avg}}$, ${\mathrm{SKT}}_{\mathrm{slope}}$, and ${\mathrm{SKT}}_{\mathrm{std}}$ as shown in Table 1.

### 2.3. Kernel-Based Extreme-Learning Machine (K-ELM)

In general, the weights and hidden-layer biases of traditional neural networks can be obtained using an optimization process based on iterative processes to find optimal parameters [27]. Obtaining appropriate parameters during the training period takes a long time because of the iterative process. However, an extreme-learning machine (ELM), based on single-hidden- layer feedforward neural networks (SLFNs), randomly generates the input weights and hidden-layer biases [28]. Therefore, the computation time for the training period is extremely fast and the classification accuracy is also improved.

Assuming that M class numbers, N arbitrary distinct input samples $\left\{\left(x_{i},t_{i}\right)|x_{i}\in R^{N},\;t_{i}\in R^{D},i=1,\ldots ,N\right\}$, and the number of hidden nodes *L* are given, the standard SLFNs is mathematically described as follows:

(1) $$\sum_{j=1}^{L}\beta_{j}{}^{T}h_{j}\left(x_{i}\right)\;=\;h\left(x_{i}\right)B\;=\;t_{i}{}^{T}\left(i=1,\ldots N\right),\;\beta_{j}\in R^{M}$$

where $B\;=\;{\left[\beta_{1},\beta_{2}\ldots \beta_{L}\right]}^{T}\in R^{L\times M}$ is the weight matrix connecting the output node and the *i*th hidden node. The hidden-layer output row vector is $h\left(x_{i}\right)\;=\;\left[h_{1}\left(x_{i}\right),h_{2}\left(x_{i}\right),\ldots ,h_{L}\left(x_{i}\right)\right]\in R^{L}$ with respect to the input vector *x*, and the superscript T denotes the transpose operator. One of typical hidden-layer component $h_{j}\left(x_{i}\right)$ is described as follow:

(2) $$h_{j}\left(x_{i}\right)\;=\;ℊ\left(w_{j}{}^{T}x_{i}+b_{j}\right)$$

where ℊ(·) is activation function, $w_{j}\in R^{D}$ and $b_{j}$ are input weight vector and bias in hidden node *j*. In general, the solution for the estimated weight matrix $\hat{B}$ is obtained by minimizing the approximation error. That is,

(3) $$\underset{B\in R^{L\times M}}{\min}{\left||HB-T|\right|}^{2}$$

where:

(4) $$H\;=\;\left[\begin{array}{c} h\left(x_{1}\right)\\ \vdots \\ h\left(x_{N}\right) \end{array}\right]\;=\;{\left[\begin{array}{ccc} h_{1}\left(x_{1}\right) & \cdots & h_{L}\left(x_{1}\right)\\ \vdots & \ddots & \vdots \\ h_{1}\left(x_{N}\right) & \cdots & h_{L}\left(x_{N}\right) \end{array}\right]}_{N\times L}\;\mathrm{and}\;T\;=\;{\left[\begin{array}{c} t_{1}{}^{T}\\ \vdots \\ t_{N}{}^{T} \end{array}\right]}_{N\times M}.$$

**H** is the hidden-layer output matrix with respect to input data $x_{i}$ (*i* = 1,…, N) and **T** is the target value matrix with the number of classes *M*. To obtain the weight-matrix estimate $\hat{B}$ with the minimum training error, the minimum-norm least-squares (MNLS) method was used. The solution is described as follows:

(5) $$\hat{B}\;=\;{\left(H^{T}H\right)}^{-1}H^{T}T\;=\;H^{T}{\left(HH^{T}\right)}^{-1}T\;=\;H^{†}T,$$

where $H^{†}$ is the Moore-Penrose generalized inverse of matrix **H**. There are many methods to calculate $H^{†}$, e.g., the iterative method, orthogonal projection method, and singular value decomposition [40]. In this study, we used the ridge regression theory, adding a regularization coefficient C to the diagonal of $H^{T}H$ (or $HH^{T}$). It tends to have better generalization performance and stability. For solving the problem, the optimal solution in (3) can be changed as follow:

(6) $$\underset{B\in R^{L\times M}}{\min}\;\frac{C}{2}{\left||HB-T|\right|}^{2}+\frac{1}{2}{\left||B|\right|}^{2}.$$

Eventually, the estimated weighted matrix $\hat{B}$ can be calculated as follow:

(7) $$\hat{B}\;=H^{T}(\frac{1}{C}I+HH^{T}{)}^{-1}T,$$

where **I** is the identity matrix. The solution of the output function $f_{L}\left(x\right)$ for x can be obtained as follows:

(8) $$f_{L}\left(x\right)=h\left(x\right)H^{T}{\left(\frac{1}{C}I+HH^{T}\right)}^{-1}T.$$

Given that hidden-layer vector h(x) is unknown, the kernel matrix of the ELM based on Mercer’s condition is described as follows:

(9) $$\Omega \;=\;HH^{T}:\Omega_{ij}\;=\;h\left(x_{i}\right)\cdot h\left(x_{j}\right)\;=\;k\left(x_{i},x_{j}\right).$$

where a dot (⋅) denotes the inner product. Lastly, the output function of K-ELM is represented as follows:

(10) $$f_{L}\left(x\right)\;=\;\left[k\left(x,x_{1}\right),\ldots ,k\left(x,x_{N}\right)\right]{\left(\frac{1}{C}I\;+\;\Omega \right)}^{-1}T.$$

In this paper, we used the radial basis function (RBF) kernel ($k\left(x,x^{\prime }\right)=\exp\left(-\gamma {\left||x-x^{\prime }|\right|}^{2}\right)$).

### 2.4. Cross-Validation

We acquired three physiological signals (PPG, EDA, and SKT) from the participants in each session. Before applying the preprocessing methods, we eliminated the first 35-s period of the measured signal to exclude unstable signals. We divided the processed signals into 6-s intervals (epochs), so that the total number of epochs in the mental tasks was 170 (34 epochs × 5 sessions) per subject. Then, the features in Table 1 were utilized as the input data of K-ELM. To evaluate the classifier, we conducted a leave-one-out cross-validation (LOOCV) with one observation as test data and the remaining observations as a training data set. In other words, 170 (34 epochs × 5 sessions) procedures were repeated by changing the training data set and the test data for each subject.

### 2.5. Statistical Test

We performed a one-way analysis of variance (ANOVA) with Tukey’s HSD (honestly significant difference) test and post hoc test to compare the features of physiological signals measured in each stress sessions (MIS-S, MOS-S and SES-S). In general, one-way ANOVA is a statistical tool to analyze whether mean differences between three or more independent groups are statistically significant or not. In this study, since features of three groups were calculated from physiological signals measured in three independent stress experiments, the groups can be independent each other. Furthermore, *F*-value of one-way ANOVA can be calculated by dividing the difference of groups into difference within groups. Therefore, the large *F*-value can indicate that the groups can be well distinguished. In the opposite case, the distinction between groups is ambiguous.

Tukey’s HSD is an approach dealing with the multiple comparison problem. Although the results of one-way ANOVA are statistically significant (*p* < 0.05), it is difficult to select the accurate hypothesis due to multiple comparison problem. Therefore, post hoc test step is essential. In general, Tukey’s HSD is only applicable for pairwise comparison and the number of observation should be same.

## 3. Evaluation

In this section, we exhibit the results of our experiments including the classification performance. Firstly, we show the questionnaire scores related to each session. Secondly, we exhibit not only the performance of the proposed classifier but also the effect of each feature type. Lastly, we show the results of self-organizing mapping (SOM), which is unsupervised learning that imitates visual cortex neurons. Using this mapping, we can see the clusters of artificial neurons, which can identify the relationships among the input data [41].

### 3.1. Correlations between Stress Levels and VR Videos

Table 2 represents the questionnaire scores for the three stress-inducing sessions. The highest STAI Y-1 questionnaire score is 80. For VAS, the highest score is 10. In both scales, the higher score is related to a more stressful state for the subject. Therefore, the STAI Y-1 and VAS scores in each session were associated with the stress levels that the subtraction task with VR environments was trying to induce.

### 3.2. Classification Performance

We implemented the K-ELM classifier with four different conditions: HRV + K-ELM, SC + K-ELM, SKT + K-ELM, and integrated features (IT) + K-ELM. IT combines the HRV, SC, and SKT features, as shown in Table 1. That is, four classification types were performed.

It should be noted that the classification performance depends on the selected parameters. In particular, the K-ELM performance was associated with the regularization coefficient (C) and the kernel size (γ) of the RBF kernel in (8) [42]. In this study, we set the regularization coefficient C = {$10^{-8}$, $10^{-7}$, $10^{-6}$, $10^{-5}$, $10^{-4}$, $10^{-3}$, $10^{-2}$, $10^{-1}$, 1, 10, $10^{2}$, $10^{3}$, $10^{4}$, $10^{5}$, $10^{6}$} and kernel size γ = {$10^{-8}$, $10^{-7}$, $10^{-6}$, $10^{-5}$, $10^{-4}$, $10^{-3}$, $10^{-2}$, $10^{-1}$, 1, 10, $10^{2}$, $10^{3}$, $10^{4}$, $10^{5}$, $10^{6}$} to analyze the effects of the parameters.

**Figure 4:**
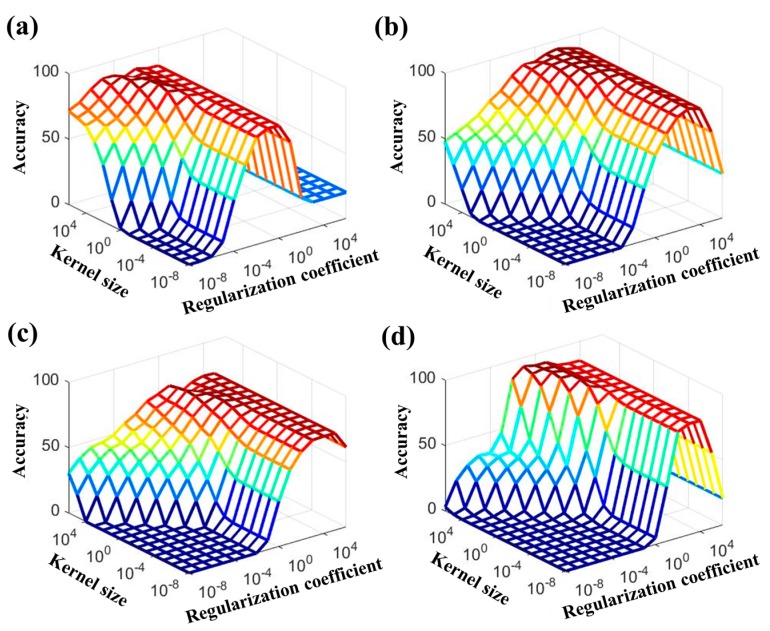
Variation of averaged accuracy with respect to the regularization coefficient and kernel size: (**a**) HRV + K-ELM; (**b**) SC + K-ELM; (**c**) SKT + K-ELM; and (**d**) IT + K-ELM.

Figure 4 indicates the mean accuracies according to the various regularization coefficients C and kernel sizes γ. The K-ELM parameters (C and γ) showing the maximum accuracy could be considered as the optimal parameters for classifying the stress levels. As shown in Figure 4, the optimal parameter values varied according to the types of features. The results showed that the optimal parameters (C, γ) were ($10^{2}$,$\;10^{-3}$), ($10^{3}$, 10), ($10^{4}$, 10), and ($10^{7}$, $10^{-1}$) in HRV + K-ELM, SC + K-ELM, SKT + K-ELM, and IT + K-ELM, respectively. Using these optimal parameters, we conducted the stress-level classification. Table 3, Table 4, Table 5 and Table 6 describe the averaged classification results for each classifier condition (HRV + K-ELM, SC + K-ELM, SKT + K-ELM, and IT + K-ELM).

Consequently, the classification accuracies of IT + K-ELM (Table 6) were generally higher than the other classification results. Considering that we utilized the same conditions except for the task-sequence order, it should be noted that we acquired high classification accuracies in both BA-S and RE-S (Table 3, Table 4, Table 5 and Table 6).

**Figure 5:**
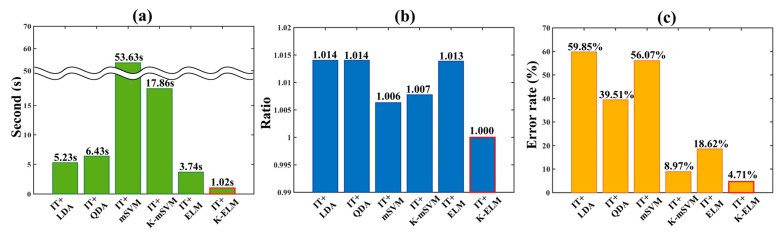
Evaluation of the proposed algorithm in comparison with results of other algorithm such as IT + LDA, IT + QDA, IT + mSVM, IT + K-mSVM and IT + ELM. (**a**) computation time; (**b**) memory usage ratio; (**c**) error rate. The red boundaries in each bar graph indicates the results of the proposed algorithm (IT + K-ELM).

**Figure 6:**
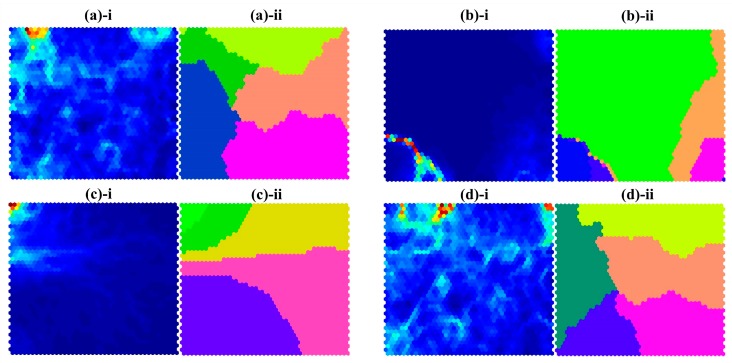
Results of self-organizing map (SOM) analysis. Each hexagon represents the neuron generated by the completive learning process. (**a**)-**i**, (**b**)-**i**, (**c**)-**i**, and (**d**)-**i** represent the neuron map trained by SOM. (**a**)-**ii**, (**b**)-**ii**, (**c**)-**ii** and (**d**)-**ii** represent the clustered neuron map made by the k-means clustering method (*k* = 5) using neuron maps. (**a**) HRV features; (**b**) SC features; (**c**) SKT features; and (**d**) IT features.

To compare the classification performances of the proposed algorithm to the those of other algorithms, we implemented linear discriminant analysis (LDA), quadratic discriminant analysis (QDA), multi-class support vector machine (mSVM), kernel-based multi-class support vector machine (K-mSVM) and extreme learning machine (ELM) [27,28,42]. Especially, implemented classification algorithms have been widely used to classify the biological information in many research fields [24,26]. However, although the deep learning algorithms (e.g., deep believe network (DBN), convolution neural network (CNN), multilayer perceptron (MLP), etc.) have excellent classification performance, we excluded them. Since conventional deep learning algorithms require heavy computation, they are difficult to be implemented in a limited environment such as microprocessor. Figure 5 describes the computation time, ratio of memory usage and error rate of each implemented classifier. Integrated feature (IT) was used for the input data and values of Figure 6 were obtained by averaging results of total 12 subjects. Especially, results of Figure 6b was calculated by dividing each memory usage of implemented classifier by memory usage of proposed algorithm (IT + K-ELM). It is because that the absolute value of memory usage depends on the computer performance. All processes were performed within the MATLAB environment with same condition.

### 3.3. Self-Organizing Map (SOM) Analysis

To investigate the organic relationships between the features calculated from the physiological signals, we applied a self-organizing map (SOM) analysis, which is unsupervised learning, to produce a two-dimensional map consisting of artificial neurons. In SOM analysis, the artificial neurons compete amongst themselves. The winning neurons can be determined by the results of the competition. We call these winning neurons SOM output neurons. These output neurons form the clusters in the map, and each cluster represents a common input pattern [43].

In this study, we trained the SOM using 2040 epochs (12 subjects × 170 epochs) from all subjects. Since the SOM input features are generated from all subjects, the clusters consisting of artificial neurons represent common input-feature patterns, contributing to the stress-level classification. Therefore, to evaluate the contributions of each feature, we made four different SOMs by changing the input features, e.g., HRV, SC, SKT, and IT.

Figure 6(a-i,b-i,c-i,d-i) shows the SOM results for each feature type: ((a) HRV, (b) SC, (c) SKT, and (d) IT). The blue regions represent that the adjacent neurons are nearby. The red regions indicate that the adjacent neurons are distant. Furthermore, to investigate whether trained neurons will build clusters or not, we applied the k-means clustering method, which classifies a given data set using a fixed number of clusters.

The results of the k-means clustering (*k* = 5) are described in Figure 6(a-ii,b-ii,c-ii,d-ii). It shows that the clustered neuron map derived by HRV features (Figure 6(a-ii)) is similar to the clustered neuron map derived by integrated features (Figure 6(d-ii)), while the other SOM results show different patterns (Figure 6(b-ii,c-ii)). In addition, it should be noted that the SOM results of HRV or integrated features (IT) show well-distributed clusters, whereas the other SOM results exhibit more irregular patterns.

### 3.4. Statistical Results

According to statistical test, seven features such as ${\mathrm{HR}}_{\mathrm{avg}}$,${\;\mathrm{NN}}_{\mathrm{avg}}$, pNN50, ${\mathrm{SCL}}_{\mathrm{avg}}$, ${\mathrm{SCR}}_{\max}$ and ${\mathrm{SKT}}_{\mathrm{avg}}$ were significantly different from each other (*p*-value < 0.05). In particular, there were features that showed statistically significant differences in all three different signals: PPG (${\mathrm{HR}}_{\mathrm{avg}}$,${\;\mathrm{NN}}_{\mathrm{avg}}$ and pNN50), EDA (${\mathrm{SCL}}_{\mathrm{avg}}$ and ${\mathrm{SCR}}_{\max}$) and SKT (${\mathrm{SKT}}_{\mathrm{avg}}$). Considering we acquired great accuracy in most classification, it may imply that measured three physiological signals in the stress situation sufficiently reflect the stress state. Furthermore, it could be a possible reason why the proposed method (IT + K-ELM) had high classification results in Table 6. The averages of *F*-values for remaining features which were not significantly different were 10.55 (PPG), 0.77 (EDA) and 2.85 (SKT), respectively. Additionally, the averages of *p*-values were lower than 0.19 (PPG), 0.47 (EDA) and 0.49 (SKT), respectively.

## 4. Discussion

**Figure 7:**
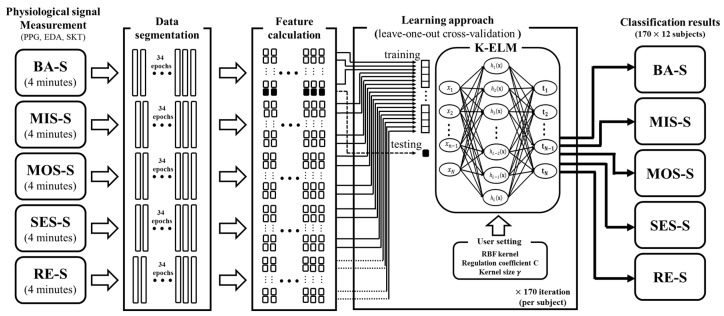
Overall feature extraction and classification process. The data-segmentation and feature-calculation steps are applied to the measured physiological signals (PPG, EDA, and SKT). The white and black boxes in the feature-calculation step represent the training and test sets, respectively.

As indicated in Figure 1, we conducted a stress-inducing experiment while simultaneously measuring several physiological signals (photoplethysmogram (PPG), electrodermal activity (EDA), and skin temperature (SKT)). Physiological signals are regulated by the autonomic nervous system (ANS), and physical or mental stress affects the activity of the ANS [3,37]. In addition, there is evidence that the characteristics of the physiological signal measured before the stress task differ from that measured after the stress task [13]. Therefore, we can expect that the stress states including two resting conditions could be well-discriminated by using the physiological signals. Accordingly, we implemented an automatic classification method to classify the stress levels. The overall feature preparation and classification processes are described in Figure 7.

To evaluate the proposed method combining kernel-based extreme learning machine (K-ELM) with various features, we followed these three steps.

Firstly, we collected data related to the subjective stress levels from the subjects (STAI Y-1 and VAS). From the results, we confirmed that the subjective stress levels were correlated with the intended stress levels in our task. For instance, most of the subjects produced low scores on MIS-S and high scores on SES-S. In other words, our VR experiment could trigger the intended stress levels, and the physiological signals were measured under the intended stress conditions. Furthermore, we performed one-way ANOVA followed by the Tukey’s HSD and post-hoc comparisons to evaluate the difference of STAI-Y1 and VAS scores from three stress stimulation. As a result, the effects of three stress stimulation are significantly different in the STAI-Y1 (*F*-value: 172.29, *p* < 0.01) and VAS (*F*-value: 274.06, *p* < 0.01), respectively.

Next, we evaluated the classification accuracies of K-ELM while changing the input features—HRV, SC, SKT, and integrated feature (IT). In general, the performance of the conventional machine-learning algorithm was governed by the corresponding parameters [27]. Since the K-ELM performance also depends on the parameters (kernel size γ and regularization coefficient C), we applied various values to K-ELM to find optimal parameters.

Figure 4 shows that the optimal parameters differ depending on the type of input feature. In other words, different parameters must be used to accurately evaluate the performances in each classification. Table 3, Table 4, Table 5 and Table 6 describe the averaged classification accuracies in HRV + K-ELM, SC + K-ELM, SKT + K-ELM, and IT + K-ELM, respectively. Except for SKT + K-ELM, the averaged classification rates of each classifier were more than 90%. In particular, the averaged classification rates of IT + K-ELM were the best (more than 95%). However, the averaged classification rates of SKT + K-ELM were approximately 77%. This implies that HRV or SC could reflect the stress-related ANS changes while SKT was not sufficiently able to show that. In fact, this result is consistent with previous studies [18,37].

It should be noted that the IT features showed the best results, which implies that the relationships among the features are also important for classifying the stress levels. The low classification results of the SKT feature might be because it is difficult to measure the core body temperature, which depends on the ANS [20].

It is also noteworthy that we successfully discriminated two resting conditions, i.e., BA-S and RE-S, even though we provided the same conditions except for the task sequences. There is evidence that it takes some time for a physiological signal to return to its original state after a stressful condition [13]. Our results could be interpreted in line with this point of view. However, high classification rates may be due to the sequence of experiments in this study. After each stress-inducing experiment, all subject had approximately 10 min of rest, but the subjects may not have been completely free from the effects of stress stimuli from previous session. This could be a limitation of our study. Therefore, in order to identify only the effect of the intended stress stimuli, it is necessary to design the experiment more elaborately, and we should use counterbalanced experiment order in future study. Furthermore, the effect of the previous stimuli on the next session might be more noticeable when a participant used a VR device for the first time. In general, unfamiliar VR environment may trigger nausea to beginners as well. Although, all subjects are approximately 25 years of age and very healthy, it might be difficult to exclude the unwanted additional stress factors. Therefore, it is possible that we have induced broader concept of stress besides our intended stress.

Figure 5 shows that the proposed method (IT + K-ELM) has the most excellent classification performance while using the minimum CPU resource allocation and memory usage during computation. In particular, computation time in Figure 5a depends on the CPU resource allocation and mathematical complexity. Among them, multi-class support vector machine (mSVM) and kernel-based multi-class support vector machine (K-mSVM) had taken quite longer computation time compared to other classifiers such as linear discriminant analysis (LDA), quadratic discriminant analysis (QDA), ELM and K-ELM. Basically, since the support vector machine (SVM) and kernel-based support vector machine (K-SVM) are binary classifier, the optimized multi-class solution can be obtained by repeatedly calculating the binary classifier problems. For instance, to obtain the k multi-class solution, binary classifiers should be calculated *k*(*k* − 1)/2 times [43]. Therefore, the mSVM and K-mSVM can use many CPU resources due to iterative process. On the other hand, although computation times of LDA and QDA are faster than mSVM and K-mSVM, the ratios of memory usage are higher than mSVM, K-mSVM, ELM and K-ELM as in Figure 5b. Basically, to obtain the discriminant hyperplane, the solutions of LDA and QDA are derived using the inverse matrix operations as well as eigenvalue and eigenvector. In particular, this process requires many memory usages [44].

In addition, computation time of ELM is higher than that of K-ELM since the solution of ELM requires the calculation of the hidden node output matrix H in (8). Therefore, whenever the number of hidden node increases, the computation time also rises. Instead, K-ELM converts from $HH^{T}$ to kernel matrix Ω in (10) and the kernel matrix Ω is determined by users. Thus, computation time of K-ELM can be reduced compared to that of ELM. Figure 5c describes that the proposed method has very small error rate despite low memory usage and short computation time. Taken together, our proposed algorithm (IT + K-ELM) is suitable for microprocessors embedded in mobile system and have excellent performance as well.

Finally, the organic relationships among the features were evaluated using self-organizing maps (SOMs). The shapes and distributions of the clusters in Figure 6(a-ii,d-ii) look very similar. This implies that the HRV was a dominant feature in the IT features, and other signals might have played some supportive roles in our classifications. In particular, the clusters in Figure 6(d-ii) were relatively well-organized into five clusters around the center of the map, in comparison with the clusters in Figure 6(a-ii). On the other hand, the shapes of the clusters in Figure 6(b-ii,c-ii) were quite disorganized and their positions were biased to one side. It implies that the SC and SKT features had an ambiguous characteristic with respect to stress-level discrimination.

It is also noteworthy that the SOM results were calculated from all subject data without group distinction. It implies that the clusters trained by the SOM algorithm exhibited characteristics reflecting the common stress levels of all subjects, and uniformed clusters indicate that the input features well represented the stress levels. According to our results, the SC and SKT features had high person-to-person variability, so the classification accuracies might be lower when we used them separately. When we integrated every feature, the classifier showed better performance, suggesting that feature integration partially improved the accuracy.

Lastly, we performed the statistical tests such as one-way ANOVA and post hoc test for investigating effects of each extracted feature in three stress sections (MIS-S, MOS-S and SES-S). As a result, a few features extracted from each physiological signal were significantly different (*p*-value < 0.05). There are features of PPG (${\mathrm{HR}}_{\mathrm{avg}}$,${\;\mathrm{NN}}_{\mathrm{avg}}$ and pNN50), EDA (${\mathrm{SCL}}_{\mathrm{avg}}$ and ${\mathrm{SCR}}_{\max}$) and SKT (${\mathrm{SKT}}_{\mathrm{avg}}$). Especially, it is known that ${\mathrm{HR}}_{\mathrm{avg}}$, ${\mathrm{NN}}_{\mathrm{avg}}$ and pNN50 can be changed whenever physiological or psychological changes of human occur [37]. Besides,${\;\mathrm{SCL}}_{\mathrm{avg}}$ and ${\mathrm{SCR}}_{\max}$ are related to the ANS activities and the changes in ANS are induced by psychological and physical stress [19]. In this experiment, since we restricted movements of the subjects, the changes in features were only triggered by stress stimulation.

In statistical analysis, the averages of *F*-values, except for statistically significant features, were 10.55 (PPG), 0.77 (EDA) and 2.85 (SKT). Especially, the average of *F*-value in PPG was higher than results of EDA and SKT. The higher *F*-value is, the more significant difference between the groups is. Therefore, the features calculated by PPG have a close relationship with the stress state. As a result, Figure 6a drawn by features of PPG was relatively well- organized into clusters compared to Figure 6b,c which were described by EDA and SKT, respectively. Besides, classification rate in Table 3 was higher than those in Table 4 and Table 5 since distinction of stress state was well expressed in features of PPG.

## 5. Conclusions

In this study, we designed a stress-inducing task using a VR device and developed a stress-level classification method using K-ELM and various features. We found that the physiological signals provided enough information about the stress level that we could classify the stress levels with over 95% accuracy. Our results showed the possibility of stress measurement using physiological signals. If we can develop a wearable device, which can more conveniently measure the physiological signals, we may utilize our proposed algorithm for stress monitoring in real-life situations as well.

## Figures and Tables

**Table 1:** All features calculated from measured physiological signals (PPG, EDA and SKT).

Measure	Description	Section
	**PPG**	
${\mathrm{HR}}_{\mathrm{avg}}$	Average heart rate	Time-domain measures
${\mathrm{NN}}_{\mathrm{avg}}$	Average NN intervals	
SDNN	Standard deviation of NN intervals	
SDSD	Standard deviation of difference between adjacent NN intervals	
RMSSD	Square root of the mean of the sum of the squares of the difference between adjacent NN intervals	
pNN20	(Number of pairs of adjacent NN intervals differing by more than 20 ms)/(total number of NN intervals)	
pNN50	(Number of pairs of adjacent NN intervals differing by more than 50 ms)/(total number of NN intervals)	
${\mathrm{LF}}_{\mathrm{normal}}$	Average of normalized low-frequency component (0.04–0.15 Hz) power	Frequency-domain measures
${\mathrm{HF}}_{\mathrm{normal}}$	Average of normalized high-frequency component (0.15–0.4 Hz) power	
LF/HF	Ratio between averages of low-frequency and high-frequency powers	
ApEn (2, 0.2)	Approximate entropy of NN intervals (*m* = 2 *r* = 0.2 × SDNN)	Nonlinear measures
SampEn (2, 0.2)	Sample entropy of NN intervals (*m* = 2 *r* = 0.2 × SDNN)	
SD1	Standard deviation of data perpendicular to the axis of line-of-identity in Poincaré plot	
SD2	Standard deviation of data along the axis of line-of-identity in Poincaré plot	
SD1/SD2	Ratio between SD1 and SD2	
	**EDA**	
${\mathrm{SC}}_{\mathrm{avg}}$	Average of total skin conductance (SC) signal	SC measure
${\mathrm{SCL}}_{\mathrm{avg}}$	Average of total SC level (SCL) signal	SCL measures
${\mathrm{SCL}}_{\mathrm{slope}}$	Difference between maximum SCL and minimum SCL	
${\mathrm{SCR}}_{\mathrm{avg}}$	Average of total SC response (SCR) signal	SCR measures
${\mathrm{SCR}}_{\max}$	Maximum SCR signal	
${\mathrm{SCR}}_{\mathrm{peak}}$	Number of peaks in the SCR signal	
	**SKT**	
${\mathrm{SKT}}_{\mathrm{avg}}$	Average of total SKT signal	SKT measures
${\mathrm{SKT}}_{\mathrm{slope}}$	Difference between maximum SKT and minimum SKT	
${\mathrm{SKT}}_{\mathrm{std}}$	Standard deviation of total SKT signal	

**Table 2:** Questionnaire score for three different mental tasks.

Subject	Age	Sex	MIS-S		MOS-S		SES-S	
			STAI	VAS	STAI	VAS	STAI	VAS
**BJY**	32	M	34	2	48	4	58	10
**KKH**	25	M	25	2	60	6	67	8
**NES**	25	F	25	2	48	6	65	10
**KSH**	28	M	28	2	64	8	71	6
**LJK**	30	M	30	2	52	4	68	6
**LSW**	25	F	25	2	68	8	71	6
**KHJ**	24	F	24	2	72	8	72	10
**SHL**	31	M	31	2	53	6	71	10
**KMH**	25	F	25	2	71	6	69	6
**JHR**	33	F	33	2	40	4	60	6
**JHM**	26	F	26	2	57	6	67	8
**JSH**	26	M	26	2	54	6	74	8
**Avg**	27.5		27.7	2.1	57.3	5.8	67.8	8

**Table 3:** Classification rates of K-ELM with HRV features (HRV + K-ELM).

	BA-S	MIS-S	MOS-S	SES-S	RE-S
BA-S	**97.55%**	0.74%	0.49%	0.98%	0.25%
MIS-S	0.00%	**92.40%**	3.43%	2.70%	1.47%
MOS-S	0.00%	3.19%	**91.67%**	3.43%	1.72%
SES-S	0.25%	1.72%	4.17%	**91.42%**	2.45%
RE-S	1.23%	2.21%	3.19%	3.68%	**89.71%**

**Table 4:** Classification rates of K-ELM with SC features (SC + K-ELM).

	BA-S	MIS-S	MOS-S	SES-S	RE-S
BA-S	**96.32% **	1.23%	0.74%	1.23%	0.49%
MIS-S	1.72%	**93.63% **	2.70%	1.96%	0.00%
MOS-S	0.25%	2.45%	**94.12% **	2.45%	0.74%
SES-S	0.98%	1.47%	3.68%	**90.93%**	2.94%
RE-S	0.25%	0.00%	1.47%	2.94%	**95.34%**

**Table 5:** Classification rates of K-ELM with SKT features (SKT + K-ELM).

	BA-S	MIS-S	MOS-S	SES-S	RE-S
BA-S	**83.09% **	7.11%	1.96%	2.70%	5.15%
MIS-S	4.66%	**74.51% **	12.50%	5.39%	2.94%
MOS-S	2.70%	9.31%	**72.06% **	10.05%	5.88%
SES-S	1.72%	5.15%	12.25%	**73.77% **	7.11%
RE-S	4.66%	3.92%	3.68%	4.41%	**83.33% **

**Table 6:** Classification rates of K-ELM with integrated features (IT + K-ELM).

	BA-S	MIS-S	MOS-S	SES-S	RE-S
BA-S	**98.53% **	0.98%	0.00%	0.25%	0.25%
MIS-S	0.00%	**95.34% **	1.72%	1.47%	1.47%
MOS-S	0.00%	2.45%	**95.10% **	2.21%	0.25%
SES-S	0.00%	0.98%	3.43%	**93.38% **	2.21%
RE-S	0.25%	1.72%	1.23%	2.70%	**94.12% **
